# Erythrocyte glucose-6-phosphate dehydrogenase deficiency-induced anaemia in children in Jos, North-Central Nigeria

**DOI:** 10.4102/ajlm.v14i1.2733

**Published:** 2025-10-14

**Authors:** Justine D. Niandat, Caroline A. Okoli

**Affiliations:** 1 Department of Medical Laboratory Science, Faculty of Health Sciences and Technology, University of Jos, Jos, Nigeria; 2 Department of Chemical Pathology, Federal Medical Centre Keffi, Keffi, Nigeria

**Keywords:** prevalence, glucose-6-phosphate dehydrogenase deficiency-induced-anaemia, children, awareness, Jos, Nigeria

## Abstract

**Background:**

Glucose-6-phosphate dehydrogenase deficiency (G6PDD), a common inherited enzyme defect, associated with severe neonatal anaemia and hyperbilirubinaemia, can result in permanent neurologic damage or death. Prevalence of G6PDD-induced anaemia in vulnerable groups, like children, is not known in our setting.

**Objective:**

This study was aimed at determining the prevalence of erythrocyte G6PDD-induced anaemia among children aged 0–5 years old seen at Jos University Teaching Hospital, North-Central Nigeria.

**Methods:**

This was a hospital-based cross-sectional study conducted from February to June 2023. Glucose-6-phosphate dehydrogenase and haemoglobin levels were analysed colourimetrically. Data were analysed; *p* < 0.05 was considered significant.

**Results:**

Out of 100 children aged 0–5 years (54 male, 46 female), 40 (40%) were G6PD deficient. Nineteen (35.2%) of the G6PD-deficient children were male and 21 (45.7%) were female. Fifty-one (51%) children were anaemic, 23 (57.5%) were G6PDD-induced; 85 (85%) of the parents had no knowledge of G6PD and its deficiency.

**Conclusion:**

This study showed a high prevalence of G6PDD-induced anaemia among children in Jos. This suggests that there may be a need for early routine G6PD screening in children for early detection and proper intervention in those with the deficiency.

**What this study adds:**

This study has objectively established high prevalence of anaemia, G6PDD and G6PDD-induced anaemia in children aged 0–5 years in Jos, Nigeria, highlighting the importance of G6PD screening in children.

## Introduction

Anaemia and glucose-6-phosphate dehydrogenase (G6PD) deficiency are causes of childhood morbidity and death, especially in malaria-endemic areas such as Nigeria. Deficiency of G6PD, an oxidoreductase that catalyses the oxidation of glucose-6-phosphate to 6-phosphogluconate or the corresponding lactose, is the most common and important enzyme deficiency in red blood cells. Mediterranean, Asian, and African G6PD deficiency variants have been reported.^1^ About 7.5% of the world’s population is estimated to have at least one G6PD deficiency variant, with 15% to 26% occurring in West Africa, and 15.3% in Nigerian children.^2,3,4^

Glucose-6-phosphate dehydrogenase is an antioxidant enzyme. It is present in all body cells, including the erythrocytes, where it helps protect the cells from damage and premature destruction by reactive species.^1^ The first step in glucose metabolism via the pentose-phosphate shunt is catalysed by G6PD, with nicotinamide adenine dinucleotide phosphate (NADPH) and ribose-5-phosphate ultimately being produced. Ribose-5-phosphate is an important component of nucleotides.^4,5^

Glucose-6-phosphate dehydrogenase deficiency is an X-linked disorder, where the enzyme deficiency occurs more in male than in female individuals, because the males only have one X chromosome when compared to the females, who have two X-chromosomes.^6^ Glucose-6-phosphate dehydrogenase deficiency is usually asymptomatic. However, certain factors, such as malaria infestation, consumption of flavin-containing foods, administration of oxidative antibiotics, or antimalarial drugs can trigger the haemolytic crisis.^1^ If not promptly diagnosed and properly managed, G6PD deficiency can cause severe hyperbilirubinaemia and its associated complications, such as anaemia, kernicterus, and even death in neonates.^7^

The World Health Organization^2^ recommends routine G6PD screening of newborns in areas where the prevalence of G6PD deficiency is as high as 3% – 5%, or more. Studies have shown that early detection reduces morbidity and mortality in newborns by establishing the aetiological diagnosis of jaundice and facilitating therapeutic choices.^1^

The prevalence of G6PD deficiency-induced anaemia among children seen in Jos University Teaching Hospital, Nigeria, has, to the best of our knowledge, not been established.^8^ Anaemia is one of the major causes of paediatric death and can affect a child’s mental, physical, and social development. With the decrease in iron levels, it can lead to poor cognitive function, thus negatively affecting a child’s academic performance, and creating further complications in later life.^9^ Thus, this study aims to determine the prevalence of G6PD deficiency-induced anaemia among children seen in Jos University Teaching Hospital. This knowledge could guide the clinician to have the right index of suspicion in children presenting with some of the clinical symptoms of the disease. This can also establish the need to include a G6PD test among the battery of tests for neonates presenting with non-specific signs and symptoms of G6PD deficiency, such as prolonged jaundice (often the first week of life), anaemia, hyperbilirubinaemia, sudden jaundice, haemoglobinuria, poor feeding, lethargy, tachycardia, and others, thus helping in the early establishment of correct diagnosis and treatment, and thereby helping to reduce the morbidity and mortality resulting from G6PD deficiency.

## Methods

### Ethical considerations

Ethical approval was sought and obtained from the ethical review committee of Jos University Teaching Hospital and Jos University Teaching Hospital Research Ethics Committee before the commencement of the study, with reference number JUTH/DCS/IREC/127XXXI/437 and registration number NHREC/JUTH/05/10/22. Data were anonymised and stored on a hard drive.

Written informed consent was obtained from the parents or legal guardians of the participants. The consent forms contained information on the study’s purpose, procedures, potential risks, benefits, confidentiality, and publishing of the study’s results.

### Study design and population

This was a hospital-based cross-sectional study. The study was carried out from 10 February to 28 June 2023 in the Paediatrics clinic, units, and wards of Jos University Teaching Hospital, located at Lamingo, Jos, in the North Local Government Area of Plateau State, North-Central Nigeria.

The minimum sample size to obtain data that is scientifically acceptable was calculated using the Thrustfield^10^ formula. The expected proportion in population based on previous studies^10^ was 8%. One hundred children aged 0 to 5 years, seen at the Paediatrics clinic, units, and wards of Jos University Teaching Hospital, and whose legal parent(s) or guardian(s) gave their assent, were recruited consecutively for this study until the required sample size was reached.

### Sample and data collection

#### Questionnaire administration

A structured questionnaire was administered to each child’s legal parent or guardian by a trained member of the research team. The questionnaires were filled by the educated parents/guardians or individually interpreted for the uneducated ones. The questionnaire contained sections that requested information on aspects of socio-demography and G6PD deficiency awareness.

#### Blood sample collection and preparation

A 2 mL venous blood sample was aseptically collected from each child by an experienced paediatrician into ethylenediaminetetraacetic acid plastic sterile vacutainer containers (BD vacutainer^®^; Becton Dickinson, Ltd., Franklin Lakes, New Jersey, United States), mixed gently, and transported to the laboratory in a cold box for immediate laboratory analysis of G6PD and erythrocyte indices.

#### Laboratory analysis

The haemoglobin concentration (g/dL), erythrocyte values (x10^12^/L) and packed cell volume (%) were obtained using an automated haematology analyser (Accu cell DX360 3parts; Shenzhen iCubio Biomedical Technology Co., Ltd, Shenzhen, China). The reference range for haemoglobin concentration for children aged ≤ 5 years is ≥11.0 g/dL.^11^ Children with a haemoglobin concentration lower than 11 g/dL were considered anaemic^12^ in this study.

Glucose 6-phosphate dehydrogenase estimation was carried out quantitatively using the ICuBio Ichaem-535 chemistry automated analyser (Shenzhen iCubio Biomedical Technology Co., Ltd, Shenzhen, China), based on the principle as explained by Pferffer et al.^6^ Glucose-6-phosphate dehydrogenase catalyses the first step in the pentose phosphate shunt, oxidising glucose-6-phosphate (G-6-P) to 6-phosphogluconate (6-PG) and reducing NADP^+^ to NADPH. The rate of formation of NADPH is proportional to the G6PD activity, and is measured as an increase in absorbance at 340 nm. Erythrocyte G6PD activity reference range was 6.97 IU/gHb – 20.5 IU/gHb (at 37°C) for children.^13^ Children with erythrocyte G6PD activity lower than 6.97 IU/gHb were considered G6PD deficient.

#### Data analysis

Data were entered into Excel spreadsheet software and analysed using the International Business Machine Statistical Package for Social Science version 26 (IBM Corp., Armonk, New York, United States). Test for normality was done using Kolmogorov-Smironv test with Lilliefors correction. Chi-square was used to analyse for the categorical variables. The *Z*-test was used to test for significance in the mean values of the numerical variables. The means for participants’ erythrocyte G6PD activity were compared using one-way analysis of variance, and *p* < 0.05 at a 95% confidence interval were considered a significant difference.

## Results

### Distribution of the study population

One hundred children participated in this study (Table 1). Two-thirds (66/100) were aged less than 1 year. There were slightly more boys (*n* = 54/100) than girls (*n* = 46/100).

**TABLE 1:** Distribution of the study population (100 children seen in Paediatrics units of Jos University Teaching Hospital, Nigeria, from April 2023 to June 2023) by age and gender.

Variable	*n*	%
**Age group (years)**		
Less than 1	66	66.0
1–3	19	19.0
3–5	15	15.0
**Gender**		
Male	54	54.0
Female	46	46.0

### Mean erythrocyte glucose-6-phosphate dehydrogenase activity of the children

Forty patients (40%) were G6PD deficient, with a mean G6PD activity of 4.07 IU/gHb ± 1.08 IU/gHb, while 60 (60%) had normal G6PD activity, with a mean of 11.58 IU/gHb ± 4.28 IU/gHb (*p <* 0.001) (Table 2).

**TABLE 2:** Mean erythrocyte glucose-6-phosphate dehydrogenase activity of 100 children seen in Paediatrics units of Jos University Teaching Hospital, Nigeria from April 2023 to June 2023.

Variable	*n*	%	Activity of erythrocyte G6PD (IU/gHb)		*p*
			Mean	s.d.	
**G6PD status**	-	-	-	-	< 0.001
G6PD deficient	40	40.0	4.07	1.08	-
G6PD normal	60	60.0	11.58	4.28	-

**Total**	**100**	**100.0**	**8.59**	**5.03**	**-**

s.d., standard deviation; G6PD, glucose-6-phosphate dehydrogenase; IU/gHb, International Unit per gram of haemoglobin.

### Erythrocyte glucose-6-phosphate dehydrogenase status distribution by gender of the children

Fifty-four (54%) of the 100 children were male. Of the 40 G6PD-deficient children, 19 (*n* = 19/40) were male (*p* = 0.29) (Table 3).

**TABLE 3:** Erythrocyte glucose-6-phosphate dehydrogenase status distribution by gender of 100 children seen in Paediatrics units of Jos University Teaching Hospital, Nigeria from April 2023 to June 2023.

Variable	G6PD deficient		G6PD normal		*p*

	*n*	%	*n*	%	
**Gender**	-	-	-	-	0.29
Male	19	35.2	35	64.8	-
Female	21	45.7	25	54.3	-

G6PD, glucose-6-phosphate dehydrogenase.

### Mean activity of erythrocyte glucose-6-phosphate dehydrogenase by age and gender of the children

Mean G6PD activity was non-significantly (*p* = 0.83) higher among the children aged 1 to 3 years compared to the other age groups, and lower (*p* = 0.29) among the female patients (7.82 IU/gHb ± 4.91 IU/gHb) compared to the male patients (9.24 IU/gHb ± 5.08 IU/gHb). Glucose-6-phosphate dehydrogenase activity was non-significantly associated with both age and gender (*F* = 0.34, *p* = 0.83 [age]; *F* = 1.13, *p* = 0.29 [gender]) (Table 4).

**TABLE 4:** The mean activity of erythrocyte glucose-6-phosphate dehydrogenase by age and gender of 100 children seen in Paediatrics units of Jos University Teaching Hospital, Nigeria from April 2023 to June 2023.

Variable	*n*	%	G6PD activity (IU/gHb)		*F*	*p*
			Mean	s.d.		
**Age (years)**	-	-	-	-	0.34	0.83
< 1	66	66.0	8.54	5.41	-	-
1–3	19	19.0	9.12	4.08	-	-
3–5	15	15.0	8.05	4.37	-	-

**Total**	**100**	**100.0**	**8.58**	**5.01**	**-**	**-**

**Gender**	-	-	-	-	1.13	0.29
Male	54	54.0	9.24	5.08	-	-
Female	46	46.0	7.82	4.91	-	-

**Total**	**100**	**100.0**	**8.58**	**5.01**	**-**	**-**

s.d., standard deviation; *F*, Fisher’s exact test; G6PD, glucose-6-phosphate dehydrogenase; IU/gHb, International Unit per gram of haemoglobin.

### Prevalence of glucose-6-phosphate dehydrogenase deficiency-induced anaemia in the children

Mean packed cell volume was lower in anaemic children (30.92% ± 6.07%) compared to non-anaemic children (34.50% ± 7.11%), which was statistically significant (*p* = 0.008). Glucose-6-phosphate dehydrogenase deficiency-induced anaemia was found in 23 (57.5%) of the children, while 28 (46.7%) of the anaemic children were non-G6PD deficiency induced. Seventeen (42.5%) of G6PD-deficient children were not anaemic and 32 (53.3%) children with normal G6PD activity had normal packed cell volume values (*p* = 0.29) (Table 5).

**TABLE 5:** Prevalence of glucose-6-phosphate dehydrogenase deficiency-induced anaemia in 100 children seen in Paediatrics units of Jos University Teaching Hospital, Nigeria, from April 2023 to June 2023.

Variable	PCV (%)		*p*	G6PD status						*p*
				G6PD deficient		G6PD normal		Total		
	Mean	s.d.		*n*	%	*n*	%	*n*	%	
**Anaemia status**	-	-	0.008	-	-	-	-	-	-	0.29
Anaemic	30.92	6.07	-	23	57.5	28	46.7	51	51.0	-
Non-anaemic	34.5	7.11	-	17	42.5	32	53.3	49	49.0	-

**Total**	**-**	**-**	**-**	**40**	**100.00**	**60**	**100.00**	**100**	**100.00**	**-**

s.d., standard deviation; G6PD, glucose-6-phosphate dehydrogenase; PCV, packed cell volume.

### Parental level of knowledge of glucose-6-phosphate dehydrogenase deficiency by glucose-6-phosphate dehydrogenase activity of children

Thirty-four (40%) children of 85 parents who had no knowledge of G6PD and its deficiency were G6PD deficient. Six children of 14 parents who had slight knowledge of G6PD were G6PD deficient (Table 6).

**TABLE 6:** Parental level of knowledge of glucose-6-phosphate dehydrogenase deficiency by glucose-6-phosphate dehydrogenase activity of children seen in Paediatrics units of Jos University Teaching Hospital, Nigeria, from April 2023 to June 2023.

Parental level of knowledge of G6PD deficiency	*n*	%	Children’s G6PD activity status			
			G6PD normal		G6PD deficient	
			*n*	%	*n*	%
No knowledge	85	85.0	51	60.00	34	40.00
Slight knowledge	14	14.0	8	57.14	6	42.90
Adequate knowledge	1	1.0	1	100.00	0	0.00

**Total**	**100**	**100.0**	**60**	**60.00**	**40**	**40.00**

G6PD, glucose-6-phosphate dehydrogenase.

## Discussion

We report the prevalence of erythrocyte G6PD deficiency-induced anaemia in children in a tertiary hospital setting in North-Central Nigeria. From this study, there was a high (40%) prevalence of G6PD deficiency, with a mean G6PD activity score of 4.07 IU/gHb ± 1.08 IU/gHb for G6PD-deficient children. Glucose-6-phosphate dehydrogenase deficiency was not age- or gender-dependent (*p* = 0.83 [age]; *p* = 0.29 [gender]). Forty (40%) of the children were anaemic and 23 (57.5%) had G6PD deficiency-induced anaemia.

The high prevalence of G6PD deficiency observed in this study is similar to that (40.7%, and mean 3.79 IU/gHb ± 1.37 IU/gHb) reported by Jatau et al. in 2019,^11^ in Jos, North-Central Nigeria, among icteric neonates. However, it is higher than the 1.50 IU/gHb ± 0.02 IU/gHb reported by Uko et al.^14^ in Calabar, South-South Nigeria. The discrepancy between the result of this study and the cited work may be because of differences in assay methods, promptness in assaying for G6PD after sample collection, and level of malaria endemicity in the geographical locations. The Nigeria Malaria Indicator Survey^15^ reported that malaria was more endemic in North-Central Nigeria compared to the South-South region of the country. The association between G6PD deficiency and malaria is particularly significant.^16^ Glucose-6-phosphate dehydrogenase-deficient individuals have a selective advantage against malaria infection, as the malaria parasite (*Plasmodium* spp.) is vulnerable to oxidative damage. The altered red blood cells in G6PD-deficient individuals create an inhospitable environment for the parasite, making it more difficult for the parasite to survive and reproduce. As a result, individuals with G6PD deficiency have a reduced risk of developing severe forms of malaria caused by *P. falciparum*. This selective advantage has contributed to a higher prevalence of G6PD deficiency in regions where malaria is endemic.^17^ Thus, it may be necessary to carry out a further study on the occurrence of malaria-induced anaemia in G6PD-deficient children in our region.

In addition, G6PD deficiency was neither age nor gender-dependent in this study. Glucose-6-phosphate dehydrogenase deficiency is an X-linked disorder, where the enzyme deficiency occurs more in male than in female individuals, because the males only have one X-chromosome when compared to the females, who have two X-chromosomes.^11^ However, from this study, more of the female patients (45.7%) were G6PD deficient (X = 7.82 IU/gHb ± 4.91 IU/gHb) compared to the males (35.2%) (X = 9.24 IU/gHb ± 5.08 IU/gHb). This agrees with the finding by Iheanacho et al. in 2017,^18^ on G6PD deficiency by gender in children at Vwang Village, Vom, Jos South, Plateau State, Nigeria, where G6PD deficiency was found to be higher in girls (23.3%) when compared to boys (19.8%). Ours, however, is contrary to the finding by Albagshi et al.,^19^ who reported a higher prevalence among male children in Eastern Saudi Arabia. Cappellini and Fiorelli, in 2008,^13^ noted that in geographical regions where the frequency of the G6PD-deficient allele is high, homozygous females are not rare. Thus, there is a need for molecular and genetic characterisation of the G6PD-deficient children within our region and beyond to provide clarity on the comprehensive aetiology of the deficiency.

Also, in this study it was observed that there was a non-significant (*p* = 0.83) increase in serum G6PD activity from 8.54 IU/gHb ± 5.41 IU/gHb in children less than 1 year to 9.12 IU/gHb ± 4.08 IU/gHb as age increased between 1 and 3 years; however, there was a decrease in the activity (8.58 IU/gHb ± 5.01 IU/gHb) with further rise in age. Age was noted not to have a significant (*F* = 0.34, *p* = 0.83) effect on G6PD activity. This implies that G6PD deficiency does not depend on age, as was also noted by Iheanacho et al.^18^ It is a genetic condition present from birth. Therefore, the G6PD activity remains relatively stable throughout a person’s lifetime.^12^

There was a high (57.5%) prevalence of G6PD-induced anaemia among children attending Jos University Teaching Hospital, Nigeria. Anaemia is one of the major causes of paediatric death and can affect a child’s mental, physical, and social development. With the decrease in iron levels, it can lead to poor cognitive function, thus negatively affecting a child’s academic performance and further complications in later life.^9^ This implies that it may be necessary to include the G6PD test as part of paediatrics routine screening tests. This will help in early detection of G6PD deficiency and in creating awareness early regarding crisis-predisposing factors such as malaria infestation, consumption of flavin-containing foods, administration of oxidative antibiotic or antimalarial drugs, and the required preventive measures. This will also aid in prompt proper treatment and case management in times of oxidative stress crisis in these subjects. Anaemia could also be caused by other factors, such as poor nutrition,^20^ as 28 (46.7%) of the anaemic children were non-G6PD deficient.

This study also showed a poor level of awareness of G6PD and its deficiency among the people, as almost 99% of them had either ‘no’ or ‘slight’ knowledge of G6PD and its deficiency before enlightenment talks from the research team. This tallies with the report of Williams et al.,^21^ who stated that there is a low level of awareness of G6PD deficiency among lay people in Nigeria.

### Limitation of the study

The limitations of this study include small sample size and recall bias, which is inherent in this type of study. Also, because of financial restrictions, we were not able to conduct a molecular analysis of G6PD. This could have helped to differentiate the G6PD genotypes.

### Conclusion

There is a high prevalence of G6PD deficiency and G6PD deficiency-induced anaemia among the children in this study, and a very poor level of awareness of this defect among the parents and guardians. This could suggest the need to screen children, especially neonates, for G6PD deficiency, to allow for early detection of this abnormality.
